# Acute effect of isometric wall squat on hemodynamic and autonomic status according to hypertensive subtypes in adults

**DOI:** 10.1007/s00421-026-06203-y

**Published:** 2026-04-22

**Authors:** Valery Duque-Villarejo, Sebastián Rosero-Cabrera, Alexandra Quiceno-Carmona, Federico Mantilla-Gómez, Juan C. Calderón, Dagnovar Aristizabal-Ocampo, Jaime Gallo-Villegas

**Affiliations:** 1 GRINMADE Group, Faculty of Medicine, University of Antioquia, Calle 51D 62-29, office 301, Manuel Uribe Ángel Building, Medellín, Colombia; 2 Physiology and Biochemistry Research Group-PHYSIS, Faculty of Medicine, University of Antioquia, Medellín, Colombia; 3 SICOR Clinical and Research Center, Medellín, Colombia

**Keywords:** Hypertension, Isometric exercise, Ambulatory blood pressure monitoring, Blood pressure, Systemic vascular resistance

## Abstract

**Purpose:**

Isometric wall squat training (IWST) is effective at lowering blood pressure (BP), primarily through reductions in total peripheral resistance (TPR). However, it remains unclear whether the effects of IWST differ according to hypertension (HTN) subtypes. This study aimed to evaluate the acute hemodynamic and autonomic responses to IWST across hypertensive subtypes in adults.

**Methods:**

Quasi-experimental study that included adults with newly diagnosed HTN, according to a 24-hour ambulatory blood pressure monitoring. The hemodynamic and autonomic responses during an IWST session (4-repetition) were evaluated using the Task Force^®^Monitor device according to their hypertensive subtypes: Isolated diastolic hypertension (IDH) and systolic-diastolic hypertension (SDH).

**Results:**

The participants (*n* = 74) had an average age of 53.0±6.8 years; 56.7% (*n* = 42) were men. None of the participants were receiving antihypertensive pharmacological treatment. Compared to SDH subtype, IDH showed a lower increase in TPR (–198.2; 95%CI:–386.4 to − 10.1 dyn·s·m²·cm^−^⁵; *p* = 0.039) but a greater increase in sympathetic activity (LF/HF ratio) (1.0; 95%CI:0.3 a 1.8; *p* = 0.008), during the averaged isometric squat repetitions. Likewise, during the averaged resting intervals, IDH subtype showed greater reductions in TPR (–181.8; 95%CI:–358.8 to − 4.7 dyn·s·m²·cm^−^⁵; *p* = 0.044) and a smaller decrease in LF/HF ratio compared to SDH subtype. Following an IWST session, both IDH and SDH subtypes exhibited significant reductions in TPR and DBP (*p* < 0.001).

**Conclusion:**

Acute responses to IWST differ by HTN subtype in untreated individuals. In particular, the IDH subtype appears to derive a more favorable peripheral vascular response. However, the intervention effectively reduced BP across subtypes, supporting its effectiveness as a low-cost, non-pharmacological strategy.

## Introduction

Hypertension (HTN) is the leading risk factor for cardiovascular disease and a major contributor to global disease burden (Forouzanfar et al. 2017; Lim et al. 2012). In 2015, it was estimated that a systolic blood pressure (SBP) of at least 110–115 mmHg accounted for the loss of 211 million disability-adjusted life years worldwide (Forouzanfar et al. 2017). Despite ongoing efforts to promote early diagnosis and implement effective treatment strategies to achieve adequate blood pressure control (Kontis et al. 2014), these targets remain far from being achieved (Collaboration 2021).

In the past 10 years, isometric exercise training has been considered an effective, practical, and safe intervention for the treatment of HTN (Edwards et al. 2023; Baffour-Awuah et al. 2023; Hansford et al. 2021). Current pathophysiological evidence suggests that isometric wall squat training (IWST)-induced blood pressure reductions may be primarily attributed to a decrease in total peripheral resistance (TPR), potentially driven by enhanced autonomic regulation of vascular tone and improved endothelial function (Edwards et al. 2024; Millar et al. 2014). Additional mechanisms may include increased baroreflex sensitivity and improved heart rate variability (HRV), reflecting favorable shifts in autonomic nervous system balance (Edwards et al. 2022b; Millar et al. 2014). Different trials and metanalysis have shown that chronic reductions in blood pressure with the IWST are approximately 11 mmHg in SBP and 5 mmHg in diastolic blood pressure (DBP) (Edwards et al. 2023; Smart et al. 2019).

While chronic adaptations to IWST are increasingly documented, acute blood pressure responses to this modality remain less well characterized. Evidence indicates that IWST elicits transient elevations in SBP and DBP during contraction phases, followed by rapid normalization and reduction post-exercise (O'Driscoll et al. 2021). The magnitude of these acute responses may vary depending on exercise intensity, individual fitness levels, and underlying cardiovascular status (Taylor et al. 2017). However, there is a notable absence of studies systematically examining whether these acute and chronic hemodynamic and autonomic responses differ across HTN subtypes.

Recent findings from our group (Aristizabal-Ocampo et al. 2023) and other researchers (Romero et al. 2013) showed that the HTN subtypes present significant hemodynamic differences in TPR, cardiac output (CO), and total arterial compliance (C_t_), which may influence the effects of both pharmacological and non-pharmacological treatments. While individuals with isolated diastolic hypertension (IDH) appear to have an elevated CO or TPR (Romero et al. 2013), those with systolic-diastolic hypertension (SDH) show a decreased or normal C_t_ and an increased TPR, which implies greater arterial dysfunction (Aristizabal-Ocampo et al. 2023). Isolated systolic hypertension (ISH) is primarily characterized by decreased C_t_, reflecting increased arterial stiffness (Aristizabal-Ocampo et al. 2023). The likely different pathophysiological mechanisms involved in different types of HTN highlight the need for more specific diagnosis and treatment approaches based on HTN subtypes (personalized medicine); moreover, the underlying hemodynamic characteristics may have prognostic implications (Medina-Lezama et al. 2018; Bourdillon et al. 2022; McEvoy et al. 2021).

To date, it remains unknown whether the individualized selection of a non-pharmacological antihypertensive treatment based on HTN subtypes or hemodynamic characteristics leads to better blood pressure control (Hanssen et al. 2022; Lu et al. 2021; Parati and Caravita 2022; McEvoy et al. 2024; Rea et al. 2018). Also, we do not know whether the acute response to isometric wall squat differs among subtypes of HTN. This study aimed to evaluate the heterogeneity of the acute effect of IWST on hemodynamic and autonomic variables according to hypertensive subtypes in patients with newly diagnosed HTN. Our hypothesis is that in patients with SDH, who present higher TPR and greater arterial dysfunction (Aristizabal-Ocampo et al. 2023; Mai et al. 2022; Choi et al. 2014), IWST may be more effective (Edwards et al. 2022b).

## Methods

### Ethics approval

The study was approved by the Research Ethics Committee of the Faculty of Medicine at the University of Antioquia in Medellín (Colombia) (Approval minutes No. 053 of September 11, 2023), in accordance with the guidelines of the 2024 Declaration of Helsinki (World Medical 2025) and Resolution No. 8430 issued by the Colombian Ministry of Health in 1993. All participants provided written informed consent.

### Study design

This was a quasi-experimental study with subgroup analyses according to the hypertensive subtypes: IDH, SDH and ISH, defined by 24-h ambulatory blood pressure monitoring (24-h ABPM) taken and analyzed in a cardiovascular-specialized center in Medellín, Colombia. A quasi-experimental study evaluates the causal effect of an intervention or manipulation on a dependent variable without randomization, while maintaining a certain degree of experimental control through temporal comparisons, non-equivalent groups, or repeated-measures designs (Des Jarlais et al. 2004).

### Participants

Participants were men and women aged between 40 and 70 years with newly diagnosed HTN confirmed by 24-h ABPM, meeting the following criteria: (i) 24-h mean blood pressure ≥ 130/80 mmHg (O'Brien et al. 2013); (ii) office blood pressure classified as grade I (SBP between 140–160 mmHg and DBP between 90–100 mmHg) (McEvoy et al. 2024; Kreutz et al. 2024); and (iii) individuals without anti-hypertensive pharmacological treatment. Exclusion criteria included musculoskeletal injuries or conditions preventing exercise performance, physical, sensory, or cognitive disabilities, a history of cardiopulmonary disease, acute or chronic inflammatory conditions, active cancer, human immunodeficiency virus (HIV) infection, diabetes mellitus, and pregnancy.

### Before the intervention

Participants were required to attend the laboratory on one occasion, abstain from food for at least 4 h before, caffeine or alcohol for 24 h before, and lastly, avoid strenuous exercise 24 h before of IWST session. Prior to the intervention, data on comorbidities and relevant medical history were collected. Insufficient physical activity was considered according to the current recommendations of the World Health Organization (Bull et al. 2020). Anthropometric assessment was performed using a Seca^®^ 2013 stadiometer (Seca, Hamburg, Germany) to measure height. Body mass and body fat percentage were determined using an Omron^®^ HBF-510LA scale (Omron Healthcare, Inc., Illinois, USA) with a precision of 0.1 kg.

Waist circumference was measured with a fiberglass anthropometric tape at the midpoint between the lower border of the last rib and the iliac crest, in a horizontal plane. Thigh circumference was measured one centimeter below the gluteal fold, perpendicular to the longitudinal axis of the right thigh.

Following the anthropometric measurements, each participant performed a preliminary 2-min isometric wall squat bout, during which the squat height was adjusted until the perceived exertion reached a rating corresponding to “difficult” (~ 7), “very difficult” (~ 8), or up to “extremely difficult” (~ 9) on the validated rating of perceived exertion (RPE) for IWST to determine the minimum tolerated knee flexion angle for training, measured with an adjustable goniometer placed on the right lower limb (Wiles et al. 2018). This perceptual criterion was explicitly informed and validated by prior work (Lea et al. 2021) and has been implemented in home-based isometric wall squat interventions (Lea et al. 2024), demonstrating that RPE is a valid and reliable indicator of intensity and effectively discriminates between knee-joint angles during this task. Based on this angle, the same position was maintained for the subsequent four training series. For example, initial testing started at a 125° knee flexion angle, and adjustments were made (increasing or decreasing the angle) to enable the participant to sustain the required duration and achieve maximal effort.

### Intervention

**Fig. 1:**
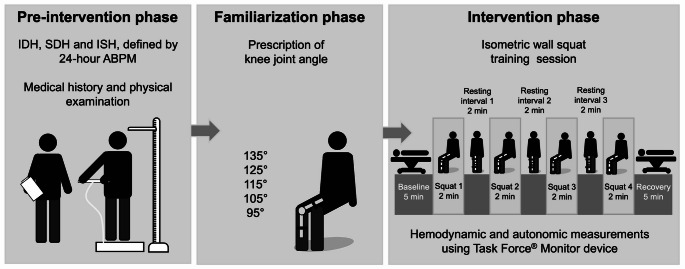
Graphical depiction of the single isometric wall squat training session. IDH, isolated diastolic hypertension; SDH, systolic-diastolic hypertension; ISH, isolated systolic hypertension; 24-hour ABPM, 24-hour ambulatory blood pressure monitoring

A supervised IWST session was conducted, where hemodynamic and autonomic variables were monitored at three time points: before (baseline), during, and after the intervention. The baseline and final recovery measurements were conducted in the supine position for five minutes. The IWST sweep consisted of four squat repetitions, each lasting two minutes, separated by two-minute standing rest intervals. A sports medicine resident supervised the session, recorded the measurements, and monitored for any adverse events (Fig. 1).

### Outcome measurements

All hemodynamic and autonomic variables, including spontaneous baroreflex sensitivity (SBS), were simultaneously recorded using the Task Force® Monitor (TFM; CNSystems Medizintechnik AG, Graz, Austria). Indices of cardiac autonomic modulation were assessed by the oscillating fluctuations in the frequency and amplitude of each R–R interval and DBP beat to beat measurement using power spectral analysis and applying an autoregressive model (Heart rate variability: standards of measurement, physiological interpretation and clinical use. Task Force of the European Society of Cardiology and the North American Society of Pacing and Electrophysiology 1996). SBS was automatically evaluated via the sequence method (Hughson et al. 1993). Continuous measurement of blood pressure was recorded by use of the vascular unloading technique at the proximal limb of the index or middle finger, which was automatically corrected to oscillometric blood pressure values obtained at the brachial artery of the contralateral arm. Heart rate (HR) was recorded through a 6-channel electrocardiogram sampled at 1 kHz and beat-to-beat stroke volume (SV) was measured with impedance cardiography (Summers et al. 2003). CO was calculated as the product of HR and SV, and TPR was estimated according to Ohm’s law. HRV and blood pressure variability parameters were computed from continuous signals using the device’s integrated software rather than from intermittently averaged measurements, thereby ensuring high temporal resolution and physiological fidelity.

The primary outcome was TPR, measured on nine moments (baseline, during each of the four squat repetitions, during each resting interval, and after the intervention). A reduction in TPR is the primary mechanism —supported by the strongest available evidence— proposed to explain the effectiveness of IWST in lowering blood pressure (Edwards et al. 2024, 2022b). Likewise, since TRP is differentially affected in hypertensive subjects, with those with SDH having a greater TRP and arterial dysfunction than those with IDH (Aristizabal-Ocampo et al. 2023), TRP is expected to be the variable most sensitive to IWST-induced changes.

Secondary outcomes included CO, cardiac index (CI), total peripheral resistance index (TPRI), SV, stroke volume index (SVI), HR, SBP, DBP, mean blood pressure (MBP), relationship between the low-frequency component of diastolic blood pressure variability and the high-frequency component of HRV (LF/HF ratio) (Heart rate variability: standards of measurement, physiological interpretation and clinical use. Task Force of the European Society of Cardiology and the North American Society of Pacing and Electrophysiology 1996), and SBS (Hughson et al. 1993). These secondary outcomes were also assessed at the same nine time points.

### Sample size

A number of 78 subjects was estimated necessary to find a mean difference in the TPR change (i.e., when comparing after vs. before the IWST sweep) between IDH and SDH of 150 dyn·s·cm^−^⁵, considering a standard deviation of 300 dyn·s·cm^−^⁵ (O’Driscoll et al. 2021), a 95% confidence level (z_1−α/2_), an 80% statistical power (z_β_), six repeated measurements (*m*), and an intraclass correlation coefficient (*ρ*) of 0.3 among the repeated measures.

### Statistical methods

The Shapiro–Wilk test was used to assess whether the distribution of quantitative variables, both in the entire sample and within subgroups, followed a normal distribution.

To describe the hypertensive groups, sociodemographic, clinical, and anthropometric variables were included. For quantitative variables with a normal distribution, mean and standard deviation were reported. For qualitative variables, the proportion of individuals exhibiting the characteristic of interest was expressed as a percentage.

Data were averaged within each predefined time points (i.e., for the 5-min baseline period; 2-min for each of the four squat bouts; 2-min for each of the three inter-set resting intervals; and for the 5-min final recovery period) for statistical comparisons. Results for all outcomes are presented as mean differences between HTN subtypes with corresponding 95% confidence intervals.

To compare between baseline and final recovery time points within each HTN group, a paired t-test was used for all variables analyzed. Analysis of covariance (ANCOVA) was then employed to compare the averaged hemodynamic and autonomic variables at final recovery periods between IDH and SDH subtypes adjusted for baseline value, age, BMI, sex, dyslipidemia, prediabetes, hypothyroidism, smoking, alcohol consumption, and physical activity level (third model). This model was chosen because the results were consistent with those obtained using an unadjusted analysis (first model), or a model only adjusted for baseline value, age, body mass index (BMI), and sex (second model).

Finally, to assess the changes in hemodynamic and autonomic variables during the squats according to hypertensive subtypes in patients with newly diagnosed HTN, a mixed-effects linear regression model (MLRM) was employed (Brown and Prescott 2015). Stata^®^ version 14.0 (StataCorp LLC, College Station, TX, USA), and IBM^®^ SPSS^®^ Statistics version 29.0 (IBM Corp., Armonk, NY, USA) were used for the analyses.

## Results

### Inclusion of participants

**Fig. 2:**
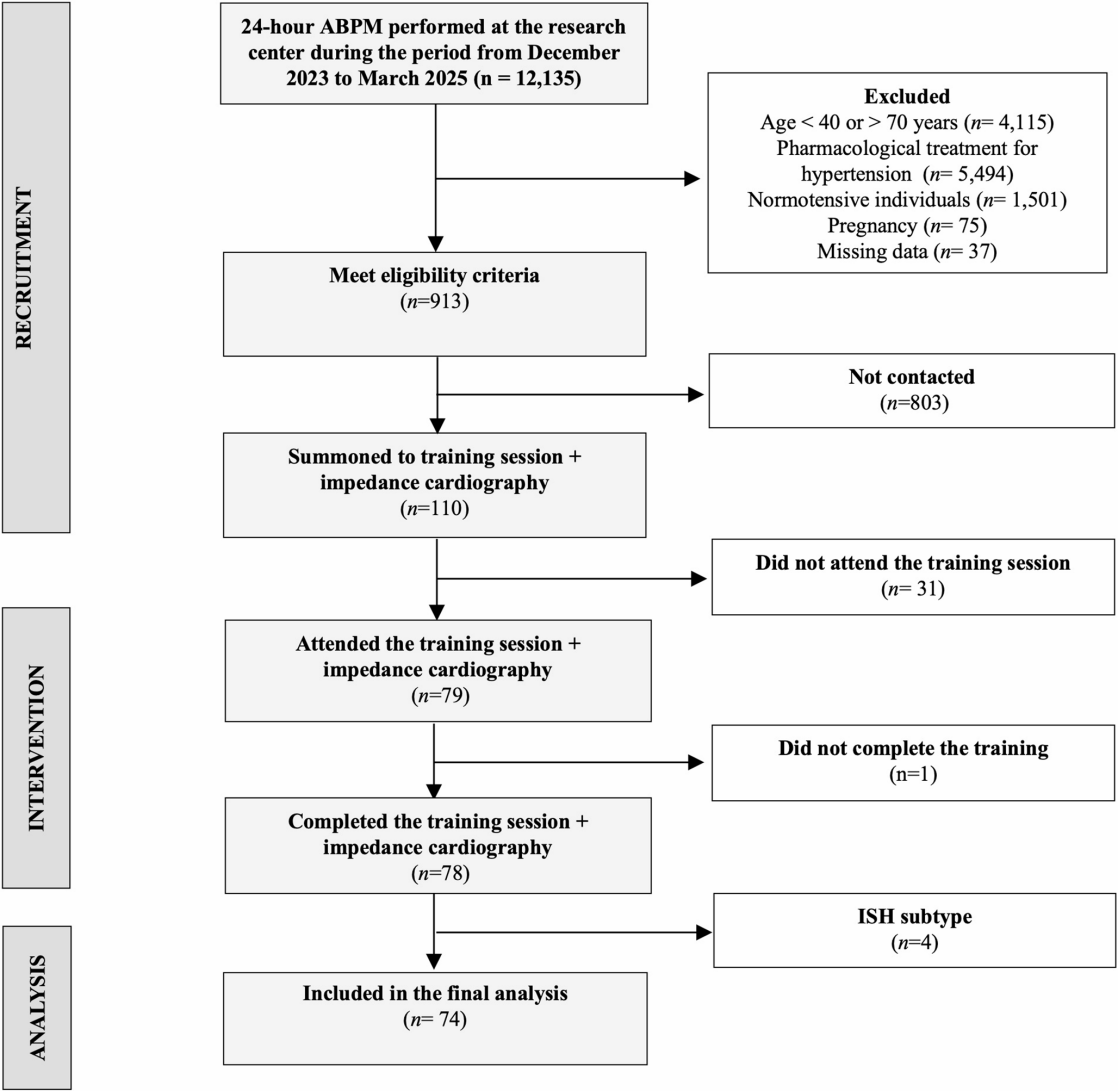
Flowchart of participant inclusion in the study. 24-hour ABPM, 24-hour ambulatory blood pressure monitoring; ISH, isolated systolic hypertension

Of 12,135 consecutive patients who underwent a 24-hour ABPM between December 2023 and March 2025, 11,222 were excluded because they were taking antihypertensive medication (*n* = 5494), were under 40 years or over 70 years old (*n* = 4115), appeared as normotensive (*n* = 1501), were pregnant women (*n* = 75), or data was missing (*n* = 37). Of the eligible patients (*n* = 913), 110 patients were contacted to schedule the IWST session with impedance cardiography; 79 attended, and only 1 did not complete the session due to symptoms prior to starting. A total of 78 participants were evaluated; however, those with the ISH subtype (*n* = 4) were excluded from the final analysis due to the small sample size. The final analysis was performed with 74 participants with IDH (*n* = 29) or SDH (*n* = 45) subtypes (Fig. 2).

### Baseline characteristics

Of the participants included in the final analysis, the mean age was 53.0 ± 6.8 years, 56.7% (*n* = 42) were men and 90.5% (*n* = 67) had a university educational level. In the SDH subtype, the average SBP and DBP were higher compared to the IDH subtype (*p* < 0.001). Similarly, the 24-hour SBP and DBP load in ABPM was higher in the SDH subtype compared to the IDH subtype (*p* < 0.001). Other sociodemographic, clinical, and functional characteristics of the participants according to their HTN subtype are presented in Table 1.

**Table 1:** Characteristics of the participants according to their hypertension subtype

Variables	Isolated diastolic hypertension (*n* = 29)	Systolic-diastolic hypertension (*n* = 45)	*P* value
	Mean ± SD	Mean ± SD	
Age (years)	51.3 ± 5.5	54.1±7.5	0.093
Weight (kg)	73.7±11.4	76.6±15.2	0.375
Stature (cm)	167.6±9.2	168.6±9.6	0.662
Body mass index (kg·m^− 2^)	26.2±3.5	26.8±3.7	0.510
Fat mass percentage (%)	32.1±8.7	30.5±9.4	0.472
Waist circumference (cm)	86.4±9.5	90.1±12.0	0.156
Proximal thigh circumference (cm)	59.6±4.6	59.2±5.1	0.678
Average 24-hour systolic blood pressure (mmHg)	124.7±3.3	135.6±4.8	< 0.001
Average 24-hour diastolic blood pressure (mmHg)	84.3±2.5	87.5±3.8	< 0.001
Average 24-hour heart rate (beat per min)	76.1±8.3	71.1±9.0	0.018
24-hour systolic load (%)	25.1±12.2	59.5±14.5	< 0.001
24-hour diastolic load (%)	61.5±14.0	72.1±13.0	< 0.001

		*n*	%	*n*	%	
Sex	Male	13	44.8%	29	64.4%	0.096
Ethnic origin	Mestizo	24	82.8%	42	93.3%	0.153
Level of education	University	25	86.2%	42	93.3%	0.306
Marital status	Single	1	3.4%	11	24.4%	0.091
	Married	26	89.7%	30	66.7%	
Socioeconomic status	Low	1	3.4%	5	11.1%	0.185
Physical activity	Insufficient	13	44.8%	24	53.3%	0.646
History of dyslipidemia	Yes	8	27.6%	17	37.8%	0.366
History of prediabetes	Yes	1	3.4%	1	2.2%	0.751
History of hypothyroidism	Yes	2	6.9%	6	13.3%	0.384
History of smoking	Current	0	0.0%	4	8.9%	0.152
Alcohol consumption intake	Current	14	48.3%	21	46.7%	0.892
Isometric squat angle	135 degrees	5	17.2%	12	26.7%	0.448
	125 degrees	15	51.7%	15	33.3%	
	115 degrees	7	24.1%	15	33.3%	
	105 degrees	2	6.9%	3	6.7%	

### Comparison between baseline and recovery periods

**Fig. 3:**
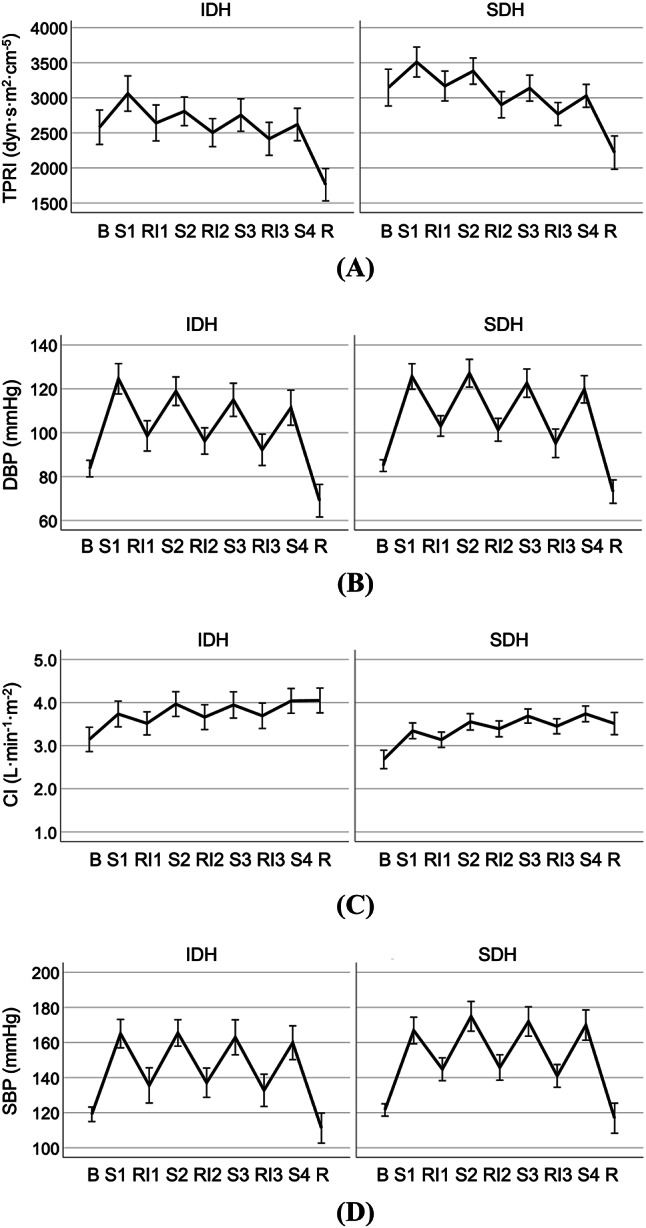
Distribution of total peripheral resistance index (**A**), diastolic blood pressure (**B**), cardiac index (**C**), and systolic blood pressure (**D**) according to hypertension subtypes during the isometric wall squat training session. IDH, isolated diastolic hypertension; SDH, systolic-diastolic hypertension; TPRI, total peripheral resistance index; DBP, diastolic blood pressure; CI, cardiac index; SBP, systolic blood pressure; B, baseline period; S, squat repetitions; RI, resting interval; R, recovery period. Error bars represent the mean with its 95% confidence interval

Columns labelled a and b of Table 2 show that for both the SDH and IDH subtypes there were decreases in TPR (*p* < 0.001), TPRI (*p* < 0.001) (Fig. 3A), DBP (*p* < 0.001) (Fig. 3B), MBP (*p* < 0.002), while HR (*p* < 0.001), SV (*p* < 0.001), SVI (*p* < 0.001), CO (*p* < 0.001), and CI (*p* < 0.001) (Fig. 3C), increased after the four squat bouts. The SDH subtype exhibited a reduction in SBS (*p* = 0.017), which was not observed in the IDH subtype (*p* = 0.053) after IWST session. No differences were found in SBP (Fig. 3D), and LF/HF ratio between before and after IWST session in either subtype of HTN.

**Table 2:** Comparison of hemodynamic and autonomic variables between hypertension subtypes before and after an isometric wall squat training session

Outcomes	Isolated diastolic hypertension (*n* = 29)^a^				*P* value^b^	Systolic-diastolic hypertension (*n* = 45)^a^				*P* value^b^	IDH^c^	SDH^c^	Absolute effect of IDH^d^	95% CI		*P* value^d^
	Before		After			Before		After			Mean adjusted	Mean adjusted				
	Mean	SD	Mean	SD		Mean	SD	Mean	SD					Lower	Upper	
Heart rate (beat per min)	74.4	11.3	85.6	14.3	< 0.001	67.1	9.7	77.0	11.7	< 0.001	80.8	80.1	0.8	-3.3	4.9	0.713
Systolic blood pressure (mmHg)	118.6	10.9	111.3	21.8	0.063	121.3	11.4	117.1	27.1	0.276	112.4	116.4	-4.1	-17.6	9.5	0.549
Diastolic blood pressure (mmHg)	83.3	9.7	68.8	18.8	< 0.001	84.4	8.6	72.8	17.2	< 0.001	69.2	72.6	-3.4	-12.9	6.1	0.479
Mean blood pressure (mmHg)	98.5	9.9	86.3	19.2	0.002	99.9	9.3	91.5	19.0	0.003	86.4	91.5	-5.0	-15.2	5.1	0.327
Stroke volume (mL)	78.5	17.4	88.3	15.6	0.002	74.9	15.5	86.0	18.6	< 0.001	86.9	86.9	0.0	-7.2	7.2	0.996
Stroke volume index (mL·m^− 2^)	43.1	8.8	48.6	9.1	0.001	40.6	9.2	46.5	10.8	< 0.001	47.2	47.4	-0.2	-4.1	3.6	0.901
Cardiac output (L·min^− 1^)	5.7	1.2	7.3	1.1	< 0.001	5.0	1.2	6.5	1.6	< 0.001	6.9	6.8	0.2	-0.4	0.7	0.549
Cardiac index (L·min^− 1^·m^− 2^)	3.1	0.7	4.0	0.8	< 0.001	2.7	0.7	3.5	0.9	< 0.001	3.8	3.7	0.1	-0.2	0.4	0.569
TPR (dyn·s·cm^− 5^)	1422.1	304.5	967.7	304.5	< 0.001	1666.2	394.8	1179.2	383.0	< 0.001	1049.9	1126.2	-76.3	-247.6	95.1	0.376
TPRI (dyn·s·m²·cm^− 5^)	2601.8	633.1	1769.9	585.4	< 0.001	3105.7	827.2	2192.9	754.0	< 0.001	1950.8	2076.3	-125.5	-425.3	174.2	0.405
LF/HF ratio	1.4	0.7	1.3	0.8	0.535	1.5	0.6	1.3	1.4	0.514	1.5	1.1	0.4	-0.1	0.9	0.156
SBS (ms/mmHg)	14.2	11.2	10.2	4.4	0.053	15.9	10.8	12.5	8.0	0.017	10.3	12.5	-2.3	-7.7	1.2	0.195

SD, standard deviation; IDH: Isolated diastolic hypertension; SDH: systolic-diastolic hypertension; TPR: Total peripheral resistance; TPRI: Total peripheral resistance index; LF/HF ratio: Relationship between the low frequency component of diastolic blood pressure variability and the high-frequency component of heart rate variability; SBS: Spontaneous baroreflex sensitivity

^a^Unadjusted means and SD

^b^Paired t-test for the change after intervention

^c^Means adjusted for age, body mass index, sex, dyslipidemia, prediabetes, hypothyroidism, smoking, alcohol consumption, physical activity level, and baseline value using ANCOVA

^d^Difference between IDH and SDH adjusted means, and ANCOVA p value for the difference between IDH and SDH adjusted means after intervention

When comparing the responses between the two HTN subtypes after the IWST session, no differences in the adjusted means of the hemodynamic and autonomic variables were observed (Table 2, columns labelled c and d). This comparison was performed using the third multivariable-adjusted model, as its results were consistent with those obtained from the other two models.

### During isometric wall squat

The mixed-effects linear regression model showed that IDH subtype exhibited a smaller increase in TPR (–123.5; 95% CI: − 235.0 to − 12.0 dyn·s·cm^− 5^; *p* = 0.030), TPRI (–198.2; 95% CI: − 386.4 to − 10.1 dyn·s·m²·cm^−^⁵; *p* = 0.039) (Fig. 3A), and DBP (–8.4; 95% CI: − 16.5 to − 0.3 mmHg; *p* = 0.043) (Fig. 3B), compared to SDH subtype during the isometric squat repetitions (Table 3). Regarding autonomic status, IDH subtype exhibited a greater increase in the sympathetic activity (LF/HF ratio) (1.0; 95% CI: 0.3 a 1.8; *p* = 0.008) compared to SDH subtype during the isometric squat repetitions (Table 3).

**Table 3:** Differences in hemodynamic and autonomic variables between hypertension subtypes during isometric wall squats

Outcomes	During the isometric wall squats					
	β coefficient	95% CI		*SE*	*z*	*P* value^a^
		Lower	Upper			
	Reference: systolic-diastolic hypertension					
Heart rate (beat per min) IDH	1.6	– 2.4	5.6	2.1	0.8	0.437
Systolic blood pressure (mmHg) IDH	– 4.4	– 12.2	3.4	4.0	– 1.1	0.270
Diastolic blood pressure (mmHg) IDH	– 8.4	– 16.5	– 0.3	4.1	– 2.0	0.043
Mean blood pressure (mmHg) IDH	– 6.4	– 13.7	0.9	3.7	– 1.7	0.085
Stroke volume (mL) IDH	– 1.2	– 5.5	3.1	2.2	– 0.5	0.590
Stroke volume index (mL·m^− 2^) IDH	– 1.2	– 3.4	1.1	1.2	– 1.0	0.312
Cardiac output (L·min^− 1^) IDH	0.2	– 0.2	0.6	0.2	0.8	0.403
Cardiac index (L·min^− 1^·m^− 2^) IDH	0.1	– 0.2	0.3	0.1	0.6	0.582
Total peripheral resistance (dyn·s·cm^− 5^) IDH	– 123.5	– 235.0	– 12.0	56.9	– 2.2	0.030
Total peripheral resistance index (dyn·s·m²·cm^− 5^) IDH	– 198.2	– 386.4	– 10.1	96.0	– 2.1	0.039
LF/HF ratio IDH	1.0	0.3	1.8	0.4	2.6	0.008
SBS (ms/mmHg) IDH	– 1.3	– 3.2	0.6	1.0	– 1.4	0.171

^a^Results of mixed-effects linear regression model for each hemodynamic and autonomic variable. IDH: Isolated diastolic hypertension; LF/HF ratio: Relationship between the low-frequency component of diastolic blood pressure variability and the high-frequency component of heart rate variability; SBS: Spontaneous baroreflex sensitivity

### During isometric wall squats resting intervals

IDH subtype showed greater reductions in TPR (–118.6; 95% CI: – 222.1 to – 15.0 dyn·s·cm^− 5^; *p* = 0.025), and TPRI (–181.8; 95% CI: – 358.9 to – 4.7 dyn·s·m²·cm^−^⁵; *p* = 0.044) (Fig. 3A) compared to SDH subtype during the isometric squat resting intervals (Table 4). Regarding autonomic status, the IDH subtype exhibited a lesser decrease in the LF/HF ratio (1.5; 95% CI: 0.4–2.6; *p* = 0.007) compared with the SDH subtype during the resting intervals of the isometric squat protocol (Table 4).

**Table 4:** Differences in hemodynamic and autonomic variables between hypertension subtypes during isometric wall squats resting intervals

Outcomes	Isometric wall squats resting intervals					
	β coefficient	95% CI		SE	*z*	*P* value^a^
		Lower	Upper			
	Reference: systolic-diastolic hypertension					
Heart rate (beat per min) IDH	1.4	– 2.0	4.8	1.7	0.8	0.429
Systolic blood pressure (mmHg) IDH	– 5.0	– 11.4	1.4	3.2	– 1.5	0.123
Diastolic blood pressure (mmHg) IDH	– 5.5	– 12.5	1.5	3.6	– 1.5	0.123
Mean blood pressure (mmHg) IDH	– 5.1	– 10.7	0.5	2.9	– 1.8	0.076
Stroke volume (mL) IDH	– 1.8	– 5.6	2.1	2.0	– 0.9	0.375
Stroke volume index (mL·m^− 2^) IDH	– 1.4	– 3.3	0.6	1.0	– 1.4	0.178
Cardiac output (L·min^− 1^) IDH	0.1	– 0.3	0.5	0.2	0.7	0.499
Cardiac index (L·min^− 1^·m^− 2^) IDH	0.1	– 0.2	0.3	0.1	0.5	0.622
Total peripheral resistance (dyn·s·cm^− 5^) IDH	– 118.6	– 222.1	– 15.0	52.8	– 2.2	0.025
Total peripheral resistance index (dyn·s·m²·cm^− 5^) IDH	– 181.8	– 358.9	– 4.7	90.3	– 2.0	0.044
LF/HF ratio IDH	1.5	0.4	2.6	0.5	2.7	0.007
SBS (ms/mmHg) IDH	– 1.0	– 2.8	0.8	0.9	– 1.1	0.266

^a^Results of mixed-effects linear regression model for each hemodynamic and autonomic variable. IDH: Isolated diastolic hypertension; LF/HF ratio: Relationship between the low-frequency component of diastolic blood pressure variability and the high-frequency component of heart rate variability; SBS: Spontaneous baroreflex sensitivity

## Discussion

Our study demonstrates that newly diagnosed, untreated individuals with arterial HTN exhibit heterogeneous hemodynamic and autonomic responses to IWST session according to HTN subtype. Four principal findings emerge. First, compared with the SDH subtype, individuals with IDH showed a smaller increase in DBP and TPR during isometric contractions, together with greater TPR reductions during recovery intervals and a higher LF/HF ratio during both phases. Second, following the IWST session, both subtypes exhibited increases in HR, SVI, and CI, accompanied by reductions in DBP, MBP, and TPRI. Third, only the SDH subtype demonstrated a reduction in SBS, whereas this effect was not observed in IDH. Finally, despite subtype-specific physiological responses during exercise, the overall post-exercise hemodynamic and autonomic changes were comparable between groups.

Aerobic exercise is currently recommended as part of the first-line treatment for patients with HTN (Hanssen et al. 2022; McEvoy et al. 2024; Kreutz et al. 2024), as it is effective for lowering blood pressure and decreasing the risk of complications and cardiovascular events (Pescatello et al. 2004, 2019; Fang et al. 2005; Naci et al. 2019).

The effect of exercise on blood pressure in hypertensive patients differs depending on the type of training. Aerobic resistance training decreases blood pressure by 7.6 mmHg and 4.7 mmHg for SBP and DBP, respectively. Strength training could reduce SBP by 5.7 mmHg and DBP by 5.2 mmHg; and when both aerobic resistance and strength training are combined, reductions of 5.3 mmHg in SBP and 5.6 mmHg in DBP are achieved (Hanssen et al. 2022). Likewise, the effect of exercise training is heterogeneous across blood pressure categories (Hanssen et al. 2022). For this reason, personalized exercise prescription is justified (Buford and Pahor 2012). However, it is unknown whether the hemodynamic profile or hypertensive subtype of the patients moderates this effect.

We recently reported that different HTN subtypes exhibit characteristic hemodynamic patterns (Aristizabal-Ocampo et al. 2023): (i) IDH is associated with elevated CO or TPR; (ii) SDH typically presents with reduced or normal C_t_ and increased TPR; and (iii) ISH is primarily characterized by decreased C_t_, reflecting increased arterial stiffness. Despite these differences, current clinical guidelines continue to treat “essential” HTN as a single, uniform condition, rather than recognizing it as a collection of potentially heterogeneous subgroups that may differ in prevalence, progression, and prognosis (Bourdillon et al. 2022; McEvoy et al. 2021).

Previous studies have consistently demonstrated that IWST is more effective in lowering blood pressure than other exercise modalities (Edwards et al. 2023, 2022a; O'Driscoll et al. 2022; Baffour-Awuah et al. 2023), while remains safe, easy to perform, affordable, and adaptable in intensity, becoming suitable for home-based clinical use in treating hypertensive patients (Baffour-Awuah et al. 2023). Among the mechanisms that explain the effect of IWST on blood pressure are a reduction in TPR, along with increased HRV and baroreflex sensitivity, overall likely related to improved autonomic regulation of vascular tone and enhanced endothelial function (Edwards et al. 2022b, 2024; Millar et al. 2014).

The acute physiological responses to any antihypertensive intervention form the foundation for understanding long-term adaptations. During the squat exercise, blood pressure increases primarily due to a rise in CO driven by a chronotropic response, while SV generally remains stable or decreases because of impaired venous return and increased cardiac afterload (Edwards et al. 2024). TPR tends to increase during the initial squats, followed by a stepwise reduction throughout each interval —findings that were corroborated by our results (O'Driscoll et al. 2021; Edwards et al. 2024). In a similar study conducted in 40 women (O'Driscoll et al. 2021), a significant reduction in TPR was observed post-exercise compared to baseline measurements, consistent with our findings and supporting the peripheral vascular effect of this type of intervention. During the recovery intervals, a reduction in TPR is observed, attributable to reactive hyperemia (Badrov et al. 2016). The consequent increase in shear stress serves as a mechanotransducive stimulus that promotes endothelial nitric oxide (NO) release, following the transient vascular compression induced by muscle contractions. These results reinforce the role of acute TPR reduction during IWST as a key mechanism contributing to blood pressure lowering. The novelty of our study lies in the subtype-based analysis, where we identified a more favorable peripheral vascular response in the IDH subtype compared to SDH. Although both subtypes exhibited hemodynamic benefits, our results suggest that peripheral vasoconstriction is less severe during the squat in the IDH subtype.

A possible explanation for our findings is that individuals with the SDH subtype may experience a greater degree of peripheral vascular flow restriction during IWST, possibly due to increased arterial stiffness or heightened sympathetic vasoconstrictor activity (Edwards et al. 2024, 2022b; Millar et al. 2014). This vascular load could account for the greater increase in TPR observed during the exercise session, in contrast to individuals with the IDH subtype, who may retain greater vascular flexibility or exhibit less structural and functional vascular compromise. It is possible that the greater reduction in the TPR observed in patients with the IDH subtype during resting intervals is due to enhanced NO availability and less arterial dysfunction, allowing a more effective flow-mediated hyperemic response that counteracts vasoconstrictive mechanisms (Edwards et al. 2024, 2022b; Millar et al. 2014). Myokines have been disregarded in the general model to explain the effectiveness of IWST on HTN. It is known that even a single bout of exercise induces the muscle release of myokines such as irisin or apelin, which can increase the release of NO by the endothelial cells (Han et al. 2015; Tatemoto et al. 2001; Chun et al. 2008; Son et al. 2019). Interestingly, the increase in apelin may counteract the response of the AT1R to angiotensin II (Chun et al. 2008; Wagenaar and Moll 2025). Thus, the explanatory model can be expanded to put forward that SDH subjects have a higher expression of AT1R or signaling to angiotensin II, with more constriction, inflammation, oxidative stress and less release and response to NO, which cannot be completely overcome by transient, local increases in myokines such as apelin.

The acute blood pressure and hemodynamic changes during IWST are regulated by complex interactions among central command, the exercise pressor reflex, the arterial baroreflex, and the cardiopulmonary baroreflex (Edwards et al. 2024). Regarding these cardiac and vascular autonomic responses, O’Driscoll et al. reported an increase in the R-R LF/HF ratio during squat repetitions, with a return to baseline values during the recovery period (O'Driscoll et al. 2021). Our findings are consistent with this pattern; however, we observed a heterogeneous response across subtypes, with a greater increase in the sympathetic activity in the IDH subtype compared to the SDH subtype. Nonetheless, comparing recovery revealed no significant differences in autonomic response between both subtypes. It is possible that, during the squat exercise, contraction -induced compression differentially affects group III/IV afferent fibers sensitive to mechanical stimuli in the two hypertensive subtypes. This may subsequently trigger cardio-acceleratory central command responses, characterized by increased sympathetic activation and concurrent parasympathetic withdrawal.

It is important to emphasize that the IWST intervention was equally effective to reduce DBP and MBP in IDH and SDH participants at the end of the protocol, disregarding the seemingly different mechanisms underlying the effects. From a practical perspective, these findings encourage caregivers to prescribe IWST sessions as part of the exercise routine of ambulatory individuals with elevated DBP and foster the inclusion of IWST in medical guidelines devoted to exercise prescription for health or HTN management. Also, since the effect on SDH may have been though a non-TPR mediated change indicates that other, yet clinically relevant mechanisms, are modulated by IWST, stimulating research on a wider range of HTN subtypes and potential response mechanisms. A further practical implication of the fact that IWST seems to mainly exert effects through TPR mechanisms in IDH but not on SDH can be extrapolated to other, non-exercise mediated aspects of the treatment of HTN. For instance, pharmacological and nutritional strategies mainly tackling TPR increases (e.g., use of ATR1 antagonists or beetroot supplementation) should be focused on IDH subtypes. However, according to the above hypothesis of SDH individuals being partially resistant to the IWST-mediated TPR improvements, treatment strategies for them should focus on other pathophysiological processes, such as CO or C_t_.

### Strengths and limitations

To our knowledge, this is the first study to examine the heterogeneity of the acute hemodynamic and autonomic response to IWST according to HTN subtypes. A key strength of this study is the separate and detailed analysis of hemodynamic and autonomic changes occurring during both the isometric squat and the inter-set resting periods, which allowed for a more precise characterization of the acute physiological responses. These analyses were conducted using robust statistical approaches, thereby enhancing the internal validity of the findings.

Nevertheless, several limitations should be acknowledged. First, the number of participants with ISH was low. This likely reflects the typical clinical profile of ISH, which predominantly affects older individuals with more advanced stages of hypertension who are frequently receiving antihypertensive pharmacological treatment. Consequently, the generalizability of our findings to this subgroup is limited.

Second, it is physiologically plausible that individuals with higher baseline blood pressure may exhibit a greater absolute response to IWST, given their larger margin for blood pressure reduction. However, the present study was not designed or powered to formally evaluate effect modification by baseline blood pressure levels. Accordingly, we did not perform stratified or interaction analyses based on initial blood pressure values.

In addition, indices of cardiac autonomic modulation used in this study, such as the LF/HF ratio and SBS, are indirect and non-invasive measures. While these indices are widely applied in both clinical and research settings, they do not provide a direct assessment of autonomic neural activity, which should be considered when interpreting the results.

Finally, the absence of C_t_ measurements represents another limitation. This parameter could provide complementary insights into vascular mechanics and may help refine the interpretation of the hemodynamic and autonomic responses observed during IWST. Future studies incorporating C_t_ assessment may further elucidate the vascular mechanisms underlying the differential responses among HTN subtypes.

## Conclusions

These findings indicate that the acute hemodynamic and autonomic responses to IWST differ according to HTN subtype in individuals with newly diagnosed, untreated arterial HTN. The IDH subtype appears to present a more favorable peripheral vascular response, which may justify consideration of prescription of tailored exercise and non-exercise strategies according to hemodynamic profiles.

Importantly, despite these subtype-specific differences, IWST was effective in reducing blood pressure across both subtypes, supporting its role as a simple, feasible, and non-pharmacological intervention for individuals with elevated DBP. Given its low physical demand and ease of implementation, IWST may represent a cost-effective strategy to complement early HTN management. Finally, providing patients with clear and comprehensive information regarding the physiological benefits of this intervention may enhance long-term adherence and optimize its clinical impact.

## Data Availability

Data and materials are available upon reasonable request to the corresponding author.

## Abbreviations

24-hour ABPM: 24-hour ambulatory blood pressure monitoring
ANCOVA: Analysis of covariance
BMI: Body mass index
CI: Cardiac index
CO: Cardiac output
C_t_: Total arterial compliance
DBP: Diastolic blood pressure
HIV: Human immunodeficiency virus
HR: Heart rate
HRV: Heart rate variability
HTN: Hypertension
IDH: Isolated diastolic hypertension
ISH: Isolated systolic hypertension
IWST: Isometric wall squat training
LF: Low-frequency component of diastolic blood pressure variability
HF: High-frequency component of heart rate variability
MBP: Mean blood pressure
MLRM: Mixed-effects linear regression model
NO: Nitric oxide
RPE: Rating of perceived exertion
SBP: Systolic blood pressure
SBS: Spontaneous baroreflex sensitivity
SDH: Systo-diastolic hypertension
SV: Stroke volume
SVI: Stroke volume index
TPR: Total peripheral resistance
TPRI: Total peripheral resistance index
